# Molecular Modeling of Chemosensory Protein 3 from *Spodoptera litura* and Its Binding Property with Plant Defensive Metabolites

**DOI:** 10.3390/ijms21114073

**Published:** 2020-06-06

**Authors:** Sujata Singh, Chetna Tyagi, Irfan A. Rather, Jamal S.M. Sabir, Md. Imtaiyaz Hassan, Archana Singh, Indrakant Kumar Singh

**Affiliations:** 1 Molecular Biology Research Laboratory, Deshbandhu College, University of Delhi, Kalkaji, New Delhi 110019, India; sujatasingh@db.du.ac.in; 2 Department of Microbiology, University of Szeged, Közép fasor 52, 6726 Szeged, Hungary; cheta231@gmail.com; 3 Department of Biological Sciences, Faculty of Science, King Abdulaziz University, P.O. Box80141, Jeddah 21589, Saudi Arabia; erfaan21@gmail.com (I.A.R.); jsabir2622@gmail.com (J.S.M.S.); 4 Center of Excellence in Bionanoscience Research, King Abdulaziz University, P.O. Box80141, Jeddah 21589, Saudi Arabia; 5 Centre for Interdisciplinary Research in Basic Sciences, Jamia Millia Islamia, Jamia Nagar, New Delhi 110025, India; mihassan@jmi.ac.in; 6 Department of Botany, Hans Raj College, University of Delhi, Delhi 110007, India

**Keywords:** chemosensory protein 3, DIMBOA, insect–plant interaction, *Spodoptera litura*, accelerated molecular dynamics, molecular docking

## Abstract

Chemosensory perception in insects involves a broad set of chemosensory proteins (CSPs) that identify the bouquet of chemical compounds present in the external environment and regulate specific behaviors. The current study is focused on the *Spodoptera litura* (Fabricius) chemosensory-related protein, SlitCSP3, a midgut-expressed CSP, which demonstrates differential gene expression upon different diet intake. There is an intriguing possibility that SlitCSP3 can perceive food-derived chemical signals and modulate insect feeding behavior. We predicted the three-dimensional structure of SlitCSP3 and subsequently performed an accelerated molecular dynamics (aMD) simulation of the best-modeled structure. SlitCSP3 structure has six α-helices arranged as a prism and a hydrophobic binding pocket predominated by leucine and isoleucine. We analyzed the interaction of selected host plant metabolites with the modeled structure of SlitCSP3. Out of two predicted binding pockets in SlitCSP3, the plant-derived defensive metabolites 2-b-D-glucopyranosyloxy-4-hydroxy-7-methoxy-1, 4-benzoxazin-3-one (DIMBOA), 6-Methoxy-2–benzoxazolinone (MBOA), and nicotine were found to interact preferably to the hydrophobic site 1, compared to site 2. The current study provides the potential role of CSPs in recognizing food-derived chemical signals, host-plant specialization, and adaptation to the varied ecosystem. Our work opens new perspectives in designing novel pest-management strategies. It can be further used in the development of CSP-based advanced biosensors.

## 1. Introduction

*Spodoptera litura* is a generalist lepidopteran pest with diverse host plant range around the world, including the Asian continent. *S. litura* has a polyphagous larval stage, which defoliates approximately 300 plant species [1]. It has a vast host choice, ranging from weeds and vegetables to economically significant grains and horticulture crops. Currently, substantial resources manage *S. litura* pests in the field. The contemporary scenario of pest control is achieved by synthetic chemicals, whose persistence has raised a lot of environmental, health, and food-contamination risks. Despite various efforts by an agronomist, this pest is still unmanageable [2]. This has spurred a pertinent need for alternative pest-management strategies.

The discovery of midgut-expressed chemosensory proteins (CSPs) has unlocked a new intriguing possibility of their role in perceiving, discriminating gut chemicals, and deciding insect food preferences [3]. Various behavioral and physiological aspects of herbivorous insects, such as courtship, feeding, and oviposition, are under the regulation of chemical cues released from their host plants [4]. Among those insect behaviors, foraging and settling on the suitable host plants for feeding is a complex behavior regulated by an internal mechanism. An intricate balance in insect feeding behavior, i.e., prompting foraging and feeding, is established by a myriad of midgut genes. Both compositions of insect gut microbiota and post-feeding reward systems regulate insect feeding [5,6]. Even a slight shift in the midgut system can impair balance, which leads to nutrient adversity [6]. Researchers have extensively focused on the role of oral chemosensory proteins in deciding insect food preferences. However, the role of the lower alimentary canal (gastric and intestinal) in stimulation and cessation of feeding is still elusive [7]. Identification of semiochemicals that can target insect chemosensory systems and manipulate insect behavior and physiology would be an environment-friendly insect pest-management approach [8].

Chemosensory proteins are small polypeptides (10–15 kDa) with 110–115 amino acids. They are abundantly expressed in the lymph of chemoreceptive organs and binds to different small-sized organic molecules such as semiochemicals and pheromones. CSPs are a soluble protein that transmits chemical information to the nervous system. Its 3D-folded structure is highly stable and compact, with most of its residues forming an α-helical domain [9]. CSPs have two disulfide bonds and a different binding cavity extended by tyrosine ring [10]. CSPs are expressed in diverse tissues and perform various roles. The expression of CSPs in the midgut suggests their possible role in perceiving food-derived chemical signals and regulating feeding behavior [7]. Different families of chemosensory proteins are expressed in insect sensory appendages (antennae, legs, and labial palps) and among various insect orders, such as Lepidoptera [11], Hymenoptera [12], Blattoidea [13], Hemiptera [14], Orthoptera [15] and other insect orders. In this study, SlitCSP3 was studied because of the presence of the complete protein sequence. A relatively new simulation method called accelerated molecular dynamics (aMD) [16,17] was used to model and study the dynamics of folded SlitCSP3 protein. We carried out a 500 ns long aMD simulation to achieve the stable, native-like conformation of SlitCSP3. This refined structure was docked with five plant-derived metabolites namely, 2-b-D-glucopyranosyloxy-4-hydroxy-7-methoxy-1, 4-benzoxazin-3-one (DIMBOA), 2-b-D-glucopyranosyloxy-4, 7-dimethoxy-1, 4-benzoxazin-3-one (HDMBOA), 6-Methoxy-2–benzoxazolinone MBOA, nicotine, and gossypol on two possible binding sites of SlitCSP3. The simulation of the SlitCSP3–DIMBOA docked complex was performed to see the stability of the bound complex along with their dynamic behavior. This study aimed to demystify the role of *S. litura* chemosensory protein 3 (SlitCSP3) in perceiving food-derived chemical signals.

## 2. Results

### 2.1. Sequence Alignment and 3D Modeling of SlitCSP3 Protein

**Figure 1:**
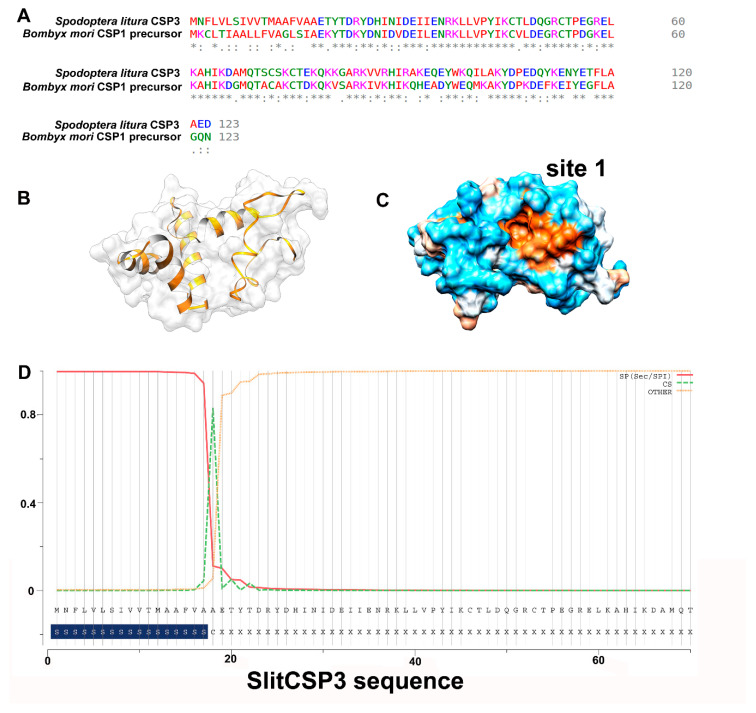
(**A**) Sequence alignment to see the conservation between SlitCSP3 and *B. mori* CSP1 precursor sequence. The sequences are well-conserved except at the N-terminus, which was not included during structure modeling. (**B**) Three-dimensional model predicted through homology modeling. (**C**) A representation of its hydrophobic surface with orange depicting the hydrophobic core. (**D**) The prediction of signal peptide sequence present in SlitCSP3 at the N-terminus till 17th residue, and 18th is the cleavage site.

For the homology modeling of *S. litura* CSP3 (SlitCSP3), a protein sequence with 123 amino acid residues was retrieved from the National Center for Biotechnology Information (NCBI) database (GenBank ID: ALJ30214.1). The obtained sequence was used to model the protein structure based on homology techniques using Modeller. The closest homology of the SlitCSP3 protein sequence (46% identity) was found with the crystal structure of chemosensory protein 1 of *Bombyx mori (Linnaeus)* [18] in the Protein Databank (PDB ID: 2JNT), and thus used as a template for structural modeling. Based on the sequence alignment of the target and template chains, the 102 amino acid residues in the long region (21–122) were modeled. However, the full sequence of template CSP1 of *B. mori* consists of 123 amino acids (GenBank ID: NP_001037065), of which the N-terminal region from 1–18 residues is the signal peptide. Their sequence alignment is shown in Figure 1A, where a very low sequence identity can be observed for the N-terminal. In contrast, a high sequence identity was observed for the rest of the alignment.

As per the parameters defined by Modeller for a reliable protein model, the parameters obtained for the modeled SlitCSP3 were the E-value of zero, GA341 score of 1.00, ModPipe Quality Score (MPQS) value of 1.4837, and zDOPE score of -1.09, and all calculated values suggested the reliability of the modeled SlitCSP3 protein structure. A cartoon representation of the modeled CSP3 structure is shown in Figure 1B, and its hydrophobic surface is represented in Figure 1C. Consequently, our analysis to check the presence of the signal peptide in SlitCSP3 suggested its presence from residues 1–17 with the cleavage site marked at the 18th residue (Figure 1D).

### 2.2. Accelerated Molecular Dynamics on the Modeled SlitCSP3

To achieve the best model, an accelerated MD was carried out, which is a bias potential function used to make the simulation “jump over” high energy barriers and to sample rare events [16,17]. An ensemble of structures was obtained after 500 ns long aMD simulation and the structures were clustered to obtain the most probable overall conformation for SlitCSP3.

**Figure 2:**
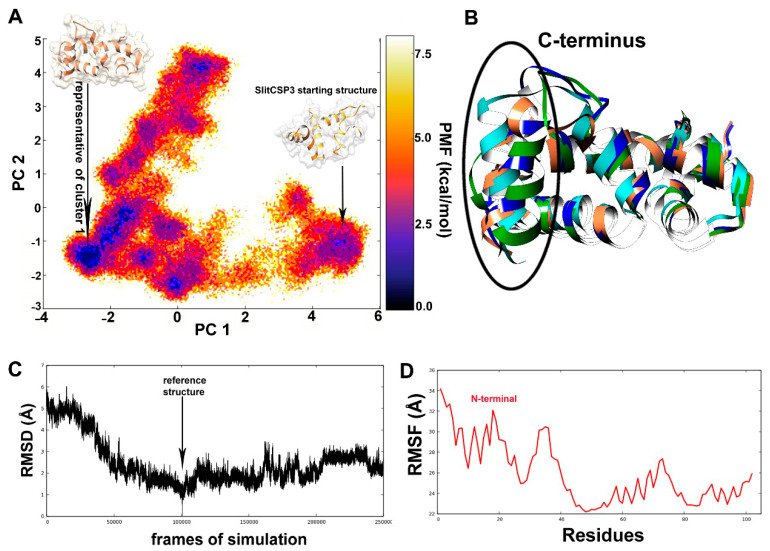
(**A**) Free energy landscape obtained from the dihedral principal component analysis of the 500 ns long accelerated MD simulation. The potential-of-mean force (PMF) stands for the change in free energy upon conformational transitions. The representative structure of cluster 1 was chosen for all further studies. (**B**) The superimposed cartoon representation of the top clusters. The major conformational differences can be seen in the C-terminus. (**C**) The root-mean-square deviation calculated for the whole trajectory to assess its stability. (**D**) The root-mean-square atomic fluctuation calculated for all residues indicates that major changes occurred at the N-terminus through its folding pathway.

The darkest blue regions of the free energy landscape (FEL) plot signify the energy minima of possible conformations, out of which the largest region denotes the first cluster (Figure 2A). A separate region denotes the incompletely folded SlitCSP3 conformation that is separated by a large energy difference (~ 5 kcal mol^−1^). The whole trajectory was clustered into the top ten clusters, out of which the representative structures of the first four clusters have been superimposed and are depicted in Figure 2B.

Figure 2C describes the root mean square deviation (RMSD) graph, the value of RMSD rapidly decreases until 50,000 steps, which show that the structures obtained during this part of the simulation were very different from the reference. There is a full dip at the 100,000th step, In the root mean square fluctuation (RMSF) graph a considerable part of the N-terminal (until 40 residues) show high fluctuation, and then again show a spike from the 60–75th residues, as shown in Figure 2D.

**Figure 3:**
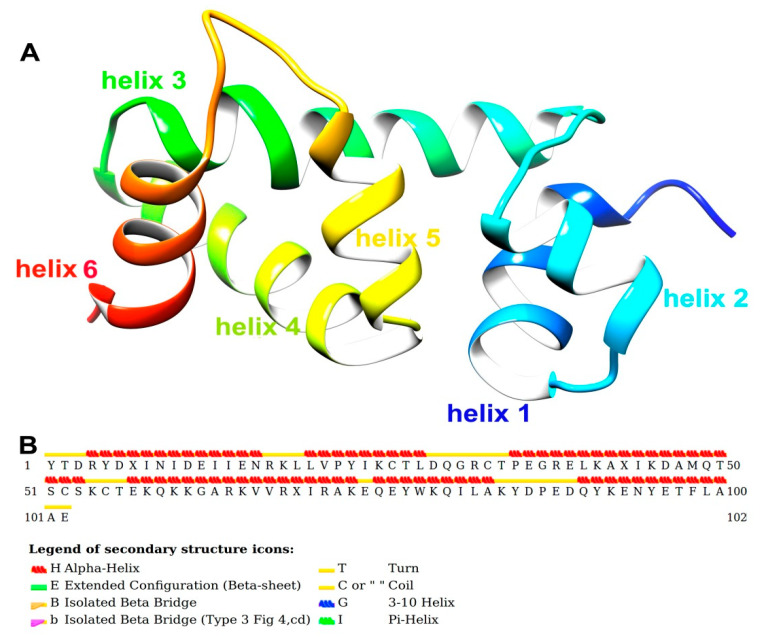
(**A**) Cartoon representation of native-like SlitCSP3 model chosen for further calculations. The structure is composed of six helices marked in different colors. (**B**) A graphical representation of STRIDE secondary structure calculation.

Figure 3A shows the post-simulated SlitCSP3 structure. The structure has helix 1 from Arg4 to Asn16, helix 2 from Leu20 to Leu28, helix 3 from Pro35 to Ser53, helix 4 from Glu57 to Ala73, helix 5 from Gln75 to Ala83, and the last helix 6 from Gln90 to Ala100. The secondary structure is depicted in Figure 3B. The secondary structural assignment clearly shows that the majority of the structure is composed of long helical regions connected by turns or coils. No extended conformations were found in the post-simulated SlitCSP3 structures.

The Ramachandran plots were obtained using RAMPAGE software (http://mordred.bioc.cam.ac.uk/~rapper/rampage.php) depicted in Supplementary Figure S1. The shaded regions define the favored (in blue) and allowed areas (in sand color). As evident from the plot, most of the residues fall in the alpha-helical region marked by black squares and triangles. Few of them fall in the extended sheet regions even though no actual beta-strands formed in the SlitCSP3 structure. The residues that form turns are typically present in the extended regions. The other residues marked with orange squares and triangles fall in the “allowed” regions, out of which three residues fall in the left-handed helical regions.

### 2.3. Identification of Binding Sites

(Computed Atlas of Surface Topography of proteins) (CASTp) and binding site module of DS 4.0 was used for predicting the binding sites of simulated SlitCSP3 protein structure. Out of ten obtained possible results, the highest-ranked binding sites, pocket 1 with a surface area of 104.680 Å^2^ and volume of 38.597 Å^3^ and pocket 2 with a surface area of 83.523 Å^2^ and volume of 36.170 Å^3^, were shortlisted. The first and second binding site residues predicted by CASTp includes Leu28, Asp29, Arg32, Lys41, Ala42, Ile44, Lys45, Gln49, Tyr77, Gln80, Ile81, Leu82, Lys84 and Gln49, Trp78, Lys79, Leu82, Ala83, Tyr85, Glu88, Asp89, Gln90, Tyr91, Asn94 respectively.

### 2.4. Interaction of SlitCSP3 with Plant-Defensive Metabolites

**Figure 4:**
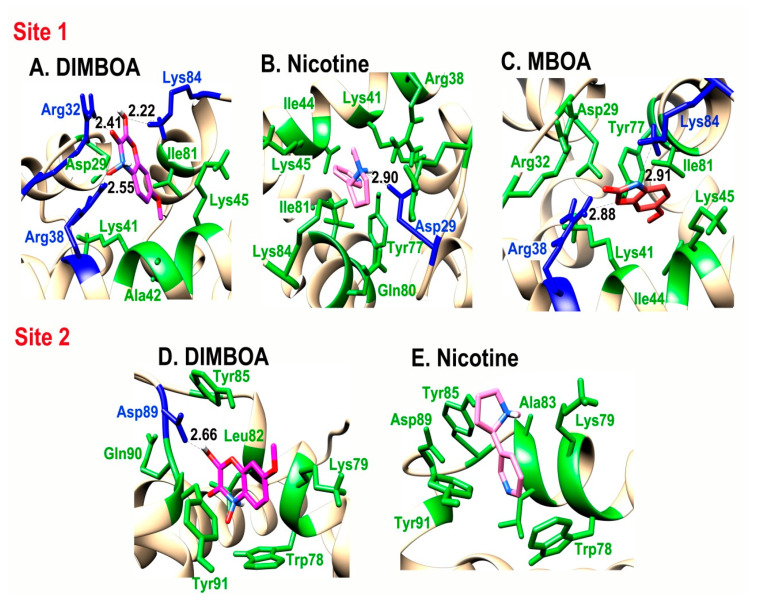
Cartoon representation of the interaction of: (**A**) DIMBOA, (**B**) Nicotine, and (**C**) MBOA at site 1, and (**D**) DIMBOA and (**E**) Nicotine at site 2 of SlitCSP3.

To confirm the interaction of preselected plant-defensive metabolites DIMBOA, HDMBOA, MBOA, gossypol, and nicotine with the post-simulated structure of SlitCSP3, a high-precision docking protocol was carried out using the Schrodinger’s glide module. Out of these five, only three ligands, namely DIMBOA, MBOA, and nicotine, were found to weakly bind with SlitCSP3 at site 1 and only DIMBOA and nicotine bound weakly at site 2. DIMBOA scored the highest with a glide score of -5.40 kcal/mol at site 1 and -5.142 kcal/mol at site 2 followed by nicotine with a glide score of -4.077 kcal/mol at site 1 and -4.838 kcal/mol at site 2. MBOA only showed binding with site 1 with a glide score of -4.214 kcal/mol. Gossypol is a bulkier molecule and would not fit in either binding site, hence it does not show any interaction. An analysis of intermolecular hydrogen bond formation between SlitCSP3 and binding ligands is indicated in Figure 4 and Table 1. The second site demonstrated low binding to selected ligands. The graphical representation of interactions at site 1 and site 2 are provided in Figure 4. 

### 2.5. Accelerated MD Simulations of SlitCSP3-Ligand Complexes

**Figure 5:**
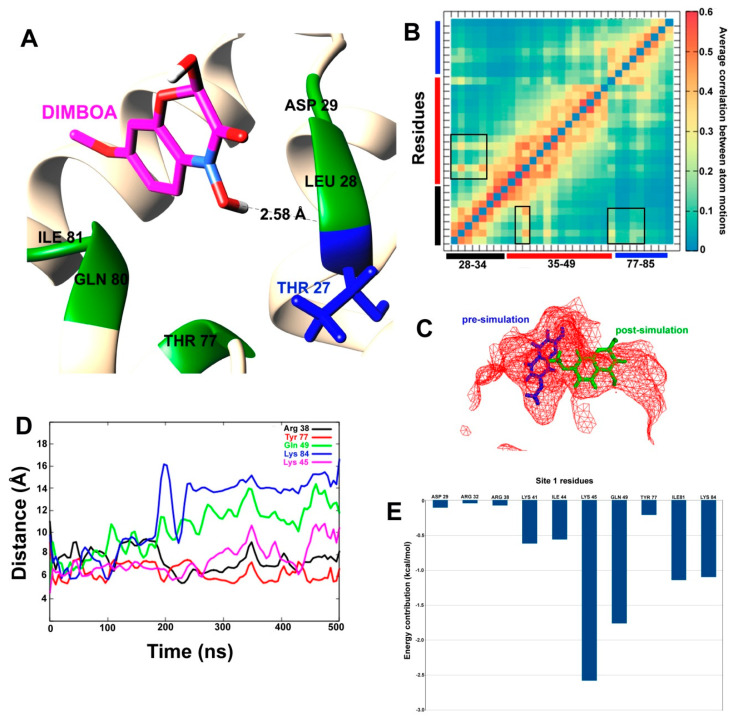
(**A**) The post-simulated interaction between DIMBOA and SlitCSP3 at site 1. (**B**) Heat map of average correlation between atomic motions calculated for site 1 amino acid residues. The black, red, and blue bars represent the three sides of the triangular binding site. The boxes highlight correlated motions between residue pairs. (**C**) The relative position of pre- and post-simulated SlitCSP3–DIMBOA complexes. (**D**) Distances (in Å) calculated for every 100th simulation frame between selected residues of site 1 and DIMBOA. (**E**) MM/PBSA-based residue-wise contribution to the binding energy.

DIMBOA-bound complexes at the two predicted sites were selected for accelerated MD simulations with weaker boost parameters, owing to the highest docking score. The post-simulation interaction between DIMBOA and SlitCSP3 at site 1 has been shown in a cartoon representation in Figure 5A. Only one hydrogen bond is formed with Thr27, while Leu28, Asp29, Thr77, Gln80, and Ile81 form hydrophobic interactions. To confirm whether binding with DIMBOA has any conformational effect on the SlitCSP3 binding site 1, we calculated the average correlation between atom motions, as shown in Figure 5B. The correlated motions have been marked by square-shaped highlights with the three bars (in black, red and blue), representing the three sides of the triangular-shaped site. It shows slight expulsion of DIMBOA from the binding site cavity (Figure 5C). To confirm this, we also calculated distances (in Å) as a function of simulation time between certain residues of the binding site and the C atom of DIMBOA (Figure 5D).

**Figure 6:**
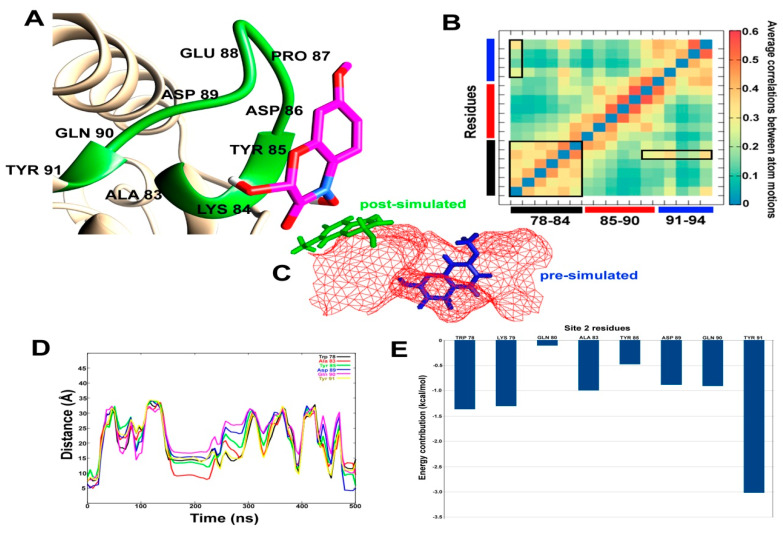
(**A**) The post-simulated interaction between DIMBOA and SlitCSP3 at site 2. (**B**) Heat map of average correlation between atomic motions calculated for site 2 amino acid residues. The black, red, and blue bars represent the three sides of the loop region. The boxes highlight correlated motions between residue pairs. (**C**) The relative position of pre- and post-simulated SlitCSP3–DIMBOA complexes. (**D**) Distances (in Å) calculated for every 100th simulation frame between selected residues of site 2 and DIMBOA. (**E**) MM/PBSA-based residue-wise contribution to the binding energy.

To analyze the binding affinity of DIMBOA at the two sites, the Molecular Mechanics/ Poisson-Boltzmann Surface Area (MM/PBSA) calculations were reported that account for per-residue contribution in the binding (Figure 5 and Figure 6).

A similar analysis was carried out for DIMBOA bound at site 2, which includes a triangular loop region before the C-terminus. The site spans from Trp78 to Asn94. As can be seen in Figure 6A, the previously bound DIMBOA has been completely expelled from the site, which can be attributed to the large correlation between atomic motions at the binding site (in Figure 6B). For example, Ala83 and Tyr91 lie exactly opposite to each other in the 3D space and show highly correlated movement. The binding site changes its shape upon interaction with DIMBOA in such a way, that the two-ring systems of DIMBOA can no longer fit (Figure 6C). The distances calculated between DIMBOA and various site 2 residues show a remarkably similar pattern (Figure 6D). Shortly after a few nanoseconds of simulation, the distances between DIMBOA and residues Trp78, Ala83, Tyr85, Asp89, Gln90, and Tyr91 increase more than 30 Å. Although this distance seems to decrease around 200 ns and few more times, the majority of DIMBOA position seems to lie far away from the binding site 2. Figure 6E depicts the contribution of each residue in the stabilization of this system where Tyr91 shows the highest contribution followed by Trp78 and Lys79. All these residues lie away from the loop region where DIMBOA is expelled out of the cavity. The results clearly show that site 1 is the most probable binding site in SlitCSP3.

## 3. Discussion

In homology modeling, the SlitCSP3 protein sequence demonstrated the closest similarity to the crystal structure of chemosensory protein 1 of *B. mori* [18]. The N-terminal region of protein could not be modeled due to the lack of a more suitable template. The SlitCSP3 protein model obtained by Modeller was found reliable as per the validation parameters. The cartoon representation in Figure 1C states that the binding cavity where site 1 is marked is hydrophobic. There is a presence of a cleavage site at 18th residue, represented in Figure 1D. It is hypothesized that the complete 123 residue-long CSP3 is a precursor form, and the removal of signal peptide must be the functionally active form. The accelerated molecular dynamics of the modeled structure was carried out to attain a close-to-native SlitCSP3 conformation. An ensemble of structures was obtained. In the SlitCSP3 modeled structure, none of the residues falls into the disallowed or outlier regions, which validates the modeled structure. The significant structural differences were observed in C-terminal folding. The representative structure of the most populated cluster lying in the energy minimum was selected for all further studies.

The root mean square of the coordinates of each “frame” of the simulation in comparison to a reference structure was calculated. RMSD analysis observed full dip at the 100000th step, which shows that the RMSD value is zero; in other words, this frame has the same structure as our reference. The graph goes on to become relatively stable after that with just a few considerable peaks, which means that no considerably significant changes occurred in the structure during the simulation after that. The simulation is more or less stable and has reached equilibrium; or, in other words, has reached “convergence”. The RMSF graph measures the deviation of the position of a particle with respect to a reference position over time. It seems that the residues lining the first binding site at helix 4 show the high fluctuation along with the N-terminus residues. The simulated SlitCSP3 structure complements the description of the experimentally determined structure of *B. mori* CSP1 [18]. Jansen et al. described it as a structure comprised of six helices arranged into a shape of a prism, which also holds for simulated SlitCSP3. The walls of the prism are formed by pairs of helices 1–2 and helices 4–5. The hydrophobic sides are generally composed of leucine and isoleucine; this cavity formed by helices 1, 2, 4, and 5 is the possible binding site. A similarity in the fold structure of *B. mori* CSP1 was established with chemosensory proteins derived from *Mamestra brassicae*, CSPMbraA6 [19,20] and *Schistocerca gregaria* CSPsg4 [21]. It is evident that this site is a highly conserved domain of CSPs and must play a crucial functional role in binding with hydrophobic ligands.

CASTp and binding site module of DS 4.0 shortlisted two binding sites based on area and volume. The docking results of native-like SlitCSP3 with selected plant metabolites were surprising. The structural difference between DIMBOA and HDMBOA is only an extra -CH_3_ (-methyl) group present in the latter. Although, this -CH_3_ group masks the O_3_ atom that forms an H-bond with Arg38 in the case of DIMBOA. This interaction could be crucial to stabilize the overall interaction, which is lacking in the case of HDMBOA. An analysis of intermolecular hydrogen bond formation between SlitCSP3 and binding ligands indicates the importance of Arg32 and Arg38, especially in the case of DIMBOA and MBOA (Figure 4, Table 1). The second site does not contain any arginine residue, and this explains the low binding of selected ligands.

On monitoring the docked complexes, we were curious to assess their stability and dynamics. To compare the two predicted binding sites on SlitCSP3, we carried out two 500 ns long accelerated MD simulations with weaker boost parameters to capture low-energy conformations. For this, only DIMBOA-bound complexes at the two predicted sites were selected, owing to the highest docking score. The post-simulation interaction between DIMBOA and SlitCSP3 at site 1 indicates that the three sides of binding at site 1 undergo certain correlated motions upon binding with a ligand, which may explain the slight expulsion of DIMBOA from the binding site cavity. It seems that DIMBOA has shifted from its original binding site. Although, all residues, Arg38, Lys45, Gln49, Tyr77, Lys84 show considerably fluctuating distance parameters from DIMBOA, the largest fluctuations are shown by Gln49 and Lys84 after 200 ns. It indicates that DIMBOA moves further away from the helix 3 side of the binding cavity. The end distance between Gln49 and Lys84 from DIMBOA is close to 15 Å. MM-PBSA calculation clarifies that Lys45, Gln49, Ile81 and Lys84 contribute the highest to the free energy of binding with DIMBOA. All these residues engage in hydrophobic interaction while the residues that form H-bond with DIMBOA, like Arg32 and Arg38, show an insignificant contribution to the overall free energy of binding. These results indicate that robust hydrophobic interactions are crucial than H-bond formation in case of binding with SlitCSP3, which also explains the relatively low binding score [22]. The MM/PBSA was reported to perform better in calculating binding free energies than the Molecular Mechanics/Generalized Born Surface Area (MM/GBSA ) method [23].

Accelerated MD simulations of DIMBOA bound at site 2 reported complete expulsion of DIMBOA from the binding site, due to change in the shape of the binding site. Also, owing to the highest dynamic fluctuation reported in the C-terminal region, this interaction is energetically unsupported. Based on these results, it is safe to conclude that the binding of DIMBOA at site 2 is not stable, which in turn indicates that this is not a binding site of SlitCSP3.

## 4. Materials and Methods

### 4.1. Sequence Retrieval and Structure Modeling

The 123 amino acid long putative SlitCSP3 protein sequence was retrieved from the NCBI database (GenBank ID: ALJ30214.1). The initial three-dimensional coordinates for CSP3 were obtained through a homology-based structure modeling. It was carried out through the webserver of Modeller named Modweb (https://modbase.compbio.ucsf.edu/modweb/) [24]. The default “slow” PSI-BLAST [25,26] method was chosen for fold assignment with adequate sampling. The chemosensory protein 1 (CSP1) of *B. mori* (PDB ID: 2JNT) was identified as the template. Based on the model score, ModPipe quality score, expect value, and z-DOPE value, the best-modeled structure was confirmed. However, as the alignment matched the sequence length of 21–122 amino acid of the target protein to the total 102 residues long template protein, the resultant protein model constituted only 102 amino acid residues. The precursor sequence of CSP1 of *B. mori* consists of 123 amino acids (GenBank ID: NP_001037065), of which the N-terminal region from 1–18 residues is signal peptide region. A calculation was carried out on the SignalP-5.0 [27] server (http://www.cbs.dtu.dk/services/SignalP-5.0/) to confirm the presence of N-terminal signal peptide in SlitCSP3 sequence.

### 4.2. Accelerated Molecular Dynamics

To improve the quality of the modeled structure and to understand its dynamics, a 500 ns long accelerated molecular dynamics simulation (aMD) was carried out. The solvated SlitCSP3 system was prepared for aMD in six consecutive steps [28]. The pressure and temperature scaling were carried out using Berendsen barostat and Langevin thermostat, respectively. SHAKE bond length constraints were applied to all bonds involving hydrogen. A short classical MD run for 100 ns was also carried out for each aMD run to calculate the torsional and total energy boost parameters.

Following Tyagi et al. [28], for each aMD simulation, particle mesh Ewald summation (PME) was used to calculate the electrostatic interactions, while long-range interactions were calculated with a cutoff of 10.0. The simulation conditions were 300 K temperature and two fs time step. The energy and boost information recorded at 1000 time-step. The NIIF clusters of Szeged and Debrecen, Hungary were sourced for running simulations on GPUs using pmemd.cuda implementation of Amber14.

The aMD simulations required extra parameters E_dihed_, α_dihed_, E_total_ and α_total_, as provided in Equation 1:

E_dihed_ = V_avg_dihed_ + a_1_ × N_res_, α_dihed_ = a_2_ × N_res_/5 E_total_ = V_avg_total_ + b_1_ × N_atoms_, α_total_ = b_2_ × N_atoms_ (1)

where N_res_ is the number of peptide residues (102 residues) and N_atoms_ is the total number of atoms in the system, which is 15,860 in the CSP3 system. V_avg_dihed_ and V_avg_total_ are the average dihedral and total potential energies obtained from the 100 ns long classical MD run. The values of coefficients a1,a2 was chosen to be 4 kcal/mol and b1, b2 to be 0.16 kcal/mol based on a study by Pierce et al. [29].

The trajectory was clustered using ‘grcarma’ [30] based on its version of dihedral principal component analysis (PCA). The dihedral based PCA was also carried out using the cpptraj module. The phi and psi torsion angles were calculated for all the residues, and the covariance matrix was calculated, which also calculates eigenvectors. The first two principal components were reweighted by the Maclaurin series expansion method [31,32].

### 4.3. Prediction of SlitCSP3 Binding Sites

Using the online server of CASTp [33], the possible binding pockets were predicted for the three-dimensional structure of SlitCSP3. CASTp measures area and volume of predicted pockets or voids by the solvent accessible surface model (Richards’ surface) and by the molecular surface model (Connolly’s surface). Five plant metabolites were selected for studying their plausible mode of interaction with SlitCSP3, namely, 2,4-Dihydroxy-7-methoxy-2H-1, 4-benzoxazin-3(4H)-one (DIMBOA, pubchem ID: 2358), 2-Hydroxy-4, 7-dimethoxy-2H-1, 4-benzoxazin-3(4H)-one (HDMBOA, pubchem ID: 11064107), 6-Methoxy-2-benzoxazolinone or Coixol (MBOA, pubchem ID: 10772), gossypol (Pubchem ID: 3503) and nicotine (Pubchem ID: 89594).

### 4.4. Grid Generation and Docking

The docking of ligands was directed at specific sites on the target enzymes which were specified by forming a cubic grid (5 Ȧ × 5 Ȧ × 5 Ȧ) around the selected residues using the Receptor Grid Generation platform of Schrödinger’s glide module [34,35]. The five ligands were prepared for docking by 2D to 3D molecular conversion using the LigPrep [36] module while using the default OPLS3e force field. All docking calculations were carried out using the Standard Precision (SP) protocol.

### 4.5. Simulations of Docked Complexes and MM/PBSA Analysis

The docked complexes of SlitCSP3 with DIMBOA at two different binding sites were also simulated, but with lesser aggressive boost parameters to effectively capture the low energy conformations involved with ligand binding. Table 2 has a summary of these parameters and their respective energy values.

The partial charges and parameters for DIMBOA were calculated using “antechamber” [37] and “leap” tools from AmberTools18 (provided in Supplementary file 1) [38].

To analyze the stability of these complexes during aMD simulations, we carried out Molecular Mechanics/Poisson Boltzmann Surface Area (MM/PBSA) [39] calculations for estimation of the free energy of binding. It is also possible to calculate the free energy decomposition as a contribution from different amino acid residues of the target protein. The GBSA model was used, where the solvent contribution to the free energy calculations was realized using a continuum or implicit solvent model.

In this method, the free energy of binding (ΔG*_bind_*) between a protein (P) and ligand (L) to form the complex PL is given as:

Equation (2):

Δ*G_bind_* = Δ*H* − TΔ*S* ~ Δ*E_MM_* + Δ*G_sol_* – *T*Δ*S* (2)

Equation (3):

Δ*E_MM_* = Δ*E_internal_* + Δ*E_electrostatic_* + Δ*E_vdw_* (3)

Equation (4):

Δ*Gsol* = Δ*G_PB/GB_* + Δ*G_SA_* (4)

where, Δ*E_MM_* + Δ*G_sol_,* and –*T*Δ*S* are the change of the gas phase molecular mechanical energy, the solvation free energy, and the conformational entropy upon binding [23], respectively. A detailed description of the underlying method is provided by Wang et al. [40,41].

## 5. Conclusions

We have performed computer-aided structural modeling of *S. litura* gut-expressed SlitCSP3 followed by a structure-based analysis of its recognition abilities and binding preferences to different host plant-derived chemical signals. *In silico* predicted SlitCSP3 structure was close to the native structure of chemosensory proteins, complements CSPs structure predicted experimentally. Out of five selected host plant metabolites (DIMBOA, HDMBOA, MBOA, nicotine, and gossypol), DIMBOA, MBOA, and nicotine demonstrated binding at predicted site 1. However, these ligands bound weakly to the binding site. We have found site 1 to be the appropriate binding site for food-derived metabolites. The MD simulation of SlitCSP3–DIMBOA complex showed the vitality of robust hydrophobic interactions than hydrogen bond formation in binding to SlitCSP3. As the binding cavity is lined by hydrophobic sides of the helices, making it well suited to bind through the hydrophobic interaction [20,22,42]. This is the reason for the low binding score. Arg32 and Arg38, which were crucial in DIMBOA interaction, proved insignificant to the overall free energy of binding on simulation. DIMBOA and nicotine could also bind to site 2 with a low glide score. As CSPs show differential recognition abilities and binding preference, SlitCSP3 might have a different binding preference from the selected host plant metabolites of the current study. Finally, the modeled structure of SlitCSP3 could be tested for its interaction with different ligands/substrates including, pesticides, because it is expressed in the gut [43]. It could be monitored for its binding properties with lipid molecules in the blood and chemical pollutants for designing advanced biosensor chip [44]. Further experimental investigation is required to validate the SlitCSP3 binding modes and substrate specificity through different genetic and molecular techniques.

## Figures and Tables

**Table 1:** SlitCSP3 residues from site 1 and site 2 showing interactions with secondary metabolites.

Site 1	Residues Forming Hydrogen Bonds	Residues Involved in Hydrophobic Interactions
DIMBOA	Arg32, Arg38, Lys84	Asp29, Lys41, Ala42, Lys45, Ile81
MBOA	Arg38, Lys84	Asp29, Arg32, Lys41, Ile44, Lys45, Tyr77, Ile81
Nicotine	Asp29	Arg38, Lys41, Ile44, Lys45, Tyr77, Gln80, Ile81, Lys84
**Site 2**		
DIMBOA	Asp89	Trp78, Lys79, Leu82, Tyr85, Gln90, Tyr91
Nicotine	No H-bond formation	Trp78, Lys79, Ala83, Tyr85, Asp89, Tyr91

**Table 2:** The values of total potential energy, dihedral energy, their respective boost parameters b1, b2 and a1, a2 applied for aMD simulations.

Boost Parameters (kcal mol^−1^)	Folding Simulation of SlitCSP3, Iamd = 3	SlitCSP3-Site1 with DIMBOA, Iamd = 1	SlitCSP3-Site 2 with DIMBOA, iamd = 1
E_Ptot_	−48643	−62150	−62056
E_dihed_	1325	1408	1400
a1, a2	4.0	NA	NA
b1, b2	0.16	0.10	0.10

## Supplementary Materials

Supplementary Materials can be found at https://www.mdpi.com/1422-0067/21/11/4073/s1. The Ramachandran Plot were obtained using RAMPAGE software and the partial charges and parameters for DIMBOA were calculated using ‘antechamber’ & ‘leap’ tools from AmberTools18 (as Supplementary Figure S1)

## Abbreviations

CSP	Chemosensory proteins
(aMD)	Accelerated molecular dynamics
SlitCSP3	*Spodoptera litura* chemosensory protein 3
PDB	Protein Database
SlitCSP3	*Spodoptera litura* chemosensory protein 3
RMSD	Root-Mean-Square Deviation
RMSF	Root-Mean-Square Atomic Fluctuation
DIMBOA	2-b-D-glucopyranosyloxy-4-hydroxy-7-methoxy-1,4-benzoxazin-3-one
HDMBOA	2-b-D-glucopyranosyloxy-4,7-dimethoxy-1,4-benzoxazin-3-one
MBOA	6-Methoxy-2-benzoxazolinone
MPQS	ModPipe Quality Score
NCBI	National Center for Biotechnology Information
CASTp	Computed Atlas of Surface Topography of proteins
MM/PBSA	Molecular Mechanics/ Poisson-Boltzmann Surface Area
MM/GBSA	Molecular Mechanics/Generalized Born Surface Area
PCA	principal component analysis
NIIF	National Information Infrastructure Development
