# Supplementary material for: Molecular Modeling of Chemosensory Protein 3 from Spodoptera litura and Its Binding Property with Plant Defensive Metabolites
Source: Int J Mol Sci. 2020 Jun 6;21(11):4073. doi: 10.3390/ijms21114073 (PMC7312704; doi:10.3390/ijms21114073)
Supplement: Supplementary file 1 [file ijms-21-04073-s001.pdf]

Supplementary Figure S1: Ramachandran Plot

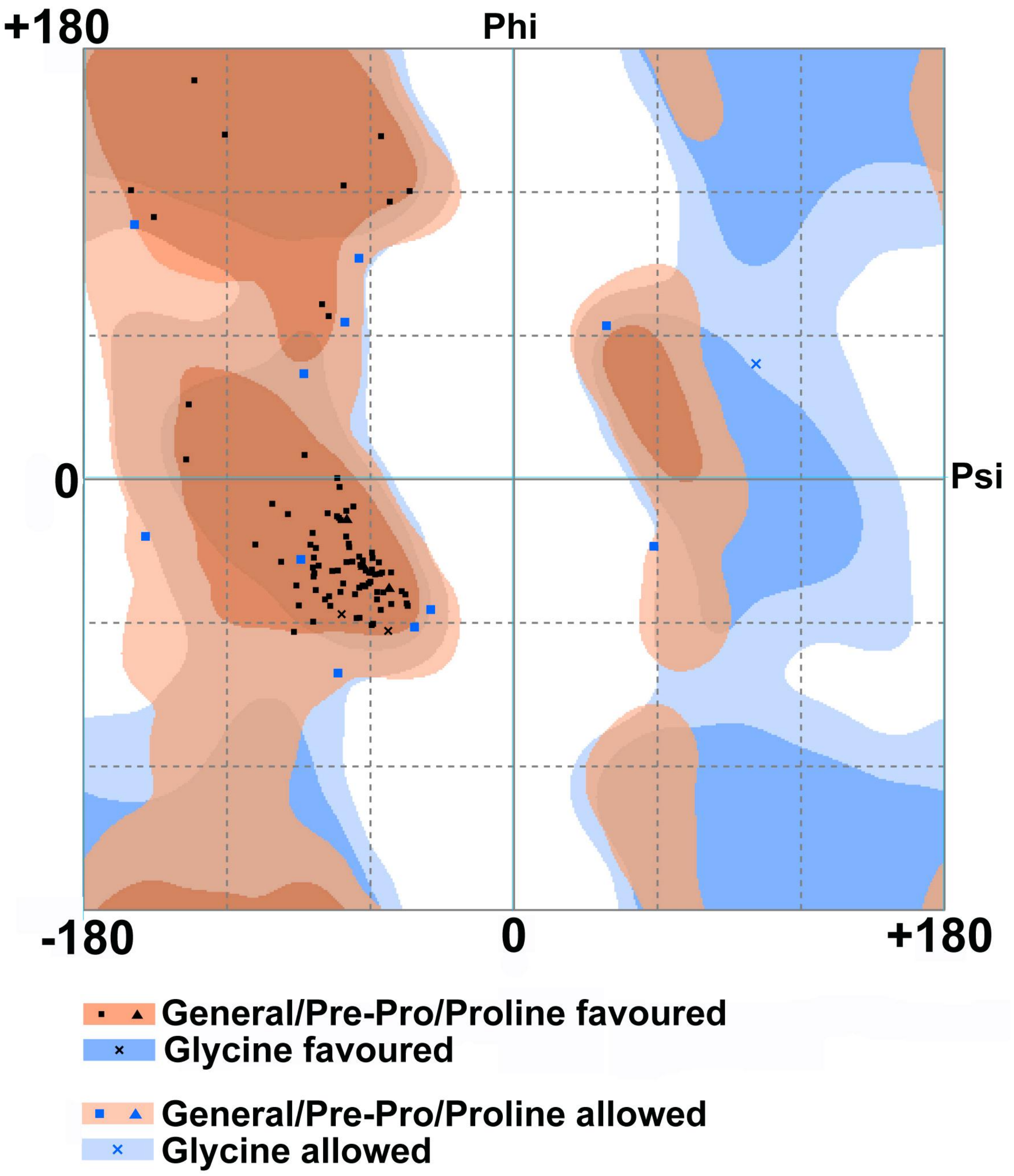

Supplementary information

The parameter file for DIMBOA in .off format which is a library format to be used with ‘tleap’ program of AmberTools18.

```
!!index array str
 "DIM"
!entry.DIM.unit.atoms table  str name  str type  int typex  int resx  int flags  int seq  int elmnt  dbl chg
 "O1" "os" 0 1 131072 1 8 -0.355900
 "O2" "oh" 0 1 131072 2 8 -0.594800
 "O3" "oh" 0 1 131072 3 8 -0.420100
 "O4" "os" 0 1 131072 4 8 -0.321900
 "O5" "o" 0 1 131072 5 8 -0.554500
 "N1" "n" 0 1 131072 6 7 -0.154500
 "C1" "ca" 0 1 131072 7 6 -0.070400
 "C2" "ca" 0 1 131072 8 6 0.139100
 "C3" "c3" 0 1 131072 9 6 0.275900
 "C4" "c" 0 1 131072 10 6 0.646500
 "C5" "ca" 0 1 131072 11 6 -0.166000
 "C6" "ca" 0 1 131072 12 6 -0.057000
 "C7" "ca" 0 1 131072 13 6 0.147100
 "C8" "ca" 0 1 131072 14 6 -0.216000
 "C9" "c3" 0 1 131072 15 6 0.112700
 "H1" "h2" 0 1 131072 16 1 0.107700
 "H2" "ha" 0 1 131072 17 1 0.170000
 "H3" "ha" 0 1 131072 18 1 0.156000
 "H4" "ha" 0 1 131072 19 1 0.146000
 "H5" "ho" 0 1 131072 20 1 0.432000
 "H6" "ho" 0 1 131072 21 1 0.435000
 "H7" "h1" 0 1 131072 22 1 0.048033
 "H8" "h1" 0 1 131072 23 1 0.048033
 "H9" "h1" 0 1 131072 24 1 0.048033
!entry.DIM.unit.atomspertinfo table  str pname  str ptype  int ptypex  int pelmnt  dbl pchg
 "O1" "os" 0 -1 0.0
 "O2" "oh" 0 -1 0.0
 "O3" "oh" 0 -1 0.0
 "O4" "os" 0 -1 0.0
 "O5" "o" 0 -1 0.0
 "N1" "n" 0 -1 0.0
 "C1" "ca" 0 -1 0.0
 "C2" "ca" 0 -1 0.0
 "C3" "c3" 0 -1 0.0
 "C4" "c" 0 -1 0.0
 "C5" "ca" 0 -1 0.0
 "C6" "ca" 0 -1 0.0
 "C7" "ca" 0 -1 0.0
 "C8" "ca" 0 -1 0.0
 "C9" "c3" 0 -1 0.0
 "H1" "h2" 0 -1 0.0
 "H2" "ha" 0 -1 0.0
 "H3" "ha" 0 -1 0.0
 "H4" "ha" 0 -1 0.0
 "H5" "ho" 0 -1 0.0
 "H6" "ho" 0 -1 0.0
 "H7" "h1" 0 -1 0.0
 "H8" "h1" 0 -1 0.0
 "H9" "h1" 0 -1 0.0
!entry.DIM.unit.boundbox array dbl
 -1.000000
 0.0
 0.0
 0.0
 0.0
!entry.DIM.unit.childsequence single int
 2
```

```
!entry.DIM.unit.connect array int
 0
 0
!entry.DIM.unit.connectivity table  int atom1x  int atom2x  int flags
 1 8 1
 1 9 1
 2 9 1
 2 20 1
 3 6 1
 3 21 1
 4 13 1
 4 15 1
 5 10 2
 6 7 1
 6 10 1
 7 8 4
 7 12 4
 8 11 4
 9 10 1
 9 16 1
 11 13 4
 11 17 1
 12 14 4
 12 18 1
 13 14 4
 14 19 1
 15 22 1
 15 23 1
 15 24 1
!entry.DIM.unit.hierarchy table  str abovetype  int abovex  str belowtype  int belowx
 "U" 0 "R" 1
 "R" 1 "A" 1
 "R" 1 "A" 2
 "R" 1 "A" 3
 "R" 1 "A" 4
 "R" 1 "A" 5
 "R" 1 "A" 6
 "R" 1 "A" 7
 "R" 1 "A" 8
 "R" 1 "A" 9
 "R" 1 "A" 10
 "R" 1 "A" 11
 "R" 1 "A" 12
 "R" 1 "A" 13
 "R" 1 "A" 14
 "R" 1 "A" 15
 "R" 1 "A" 16
 "R" 1 "A" 17
 "R" 1 "A" 18
 "R" 1 "A" 19
 "R" 1 "A" 20
 "R" 1 "A" 21
 "R" 1 "A" 22
 "R" 1 "A" 23
 "R" 1 "A" 24
!entry.DIM.unit.name single str
 "DIM"
!entry.DIM.unit.positions table  dbl x  dbl y  dbl z
 0.916000 -1.632000 -0.135000
 3.220000 -1.973000 -0.137000
 1.683000 2.482000 -0.129000
 -3.759000 -0.828000 -0.022000
 3.711000 0.658000 -0.013000
 1.448000 1.104000 -0.023000
 0.124000 0.657000 0.014000
 -0.103000 -0.722000 -0.035000
 2.159000 -1.163000 0.356000
 2.542000 0.287000 0.069000
 -1.406000 -1.222000 -0.058000
 -0.964000 1.536000 0.052000
```

```
 -2.487000 -0.344000 -0.008000
 -2.266000 1.033000 0.047000
 -4.818000 0.127000 0.023000
 2.143000 -1.270000 1.449000
 -1.579000 -2.294000 -0.113000
 -0.820000 2.612000 0.086000
 -3.061000 1.770000 0.083000
 3.648000 -2.396000 0.627000
 1.886000 2.556000 -1.077000
 -4.805000 0.699000 0.957000
 -5.759000 -0.434000 0.004000
 -4.814000 0.774000 -0.861000
!entry.DIM.unit.residueconnect table  int c1x  int c2x  int c3x  int c4x  int c5x  int c6x
 0 0 0 0 0 0
!entry.DIM.unit.residues table  str name  int seq  int childseq  int startatomx  str restype  int imagingx
 "DIM" 1 25 1 "?" 0
!entry.DIM.unit.residuesPdbSequenceNumber array int
 0
!entry.DIM.unit.solventcap array dbl
 -1.000000
 0.0
 0.0
 0.0
 0.0
!entry.DIM.unit.velocities table  dbl x  dbl y  dbl z
 0.0 0.0 0.0
 0.0 0.0 0.0
 0.0 0.0 0.0
 0.0 0.0 0.0
 0.0 0.0 0.0
 0.0 0.0 0.0
 0.0 0.0 0.0
 0.0 0.0 0.0
 0.0 0.0 0.0
 0.0 0.0 0.0
 0.0 0.0 0.0
 0.0 0.0 0.0
 0.0 0.0 0.0
 0.0 0.0 0.0
 0.0 0.0 0.0
 0.0 0.0 0.0
 0.0 0.0 0.0
 0.0 0.0 0.0
 0.0 0.0 0.0
 0.0 0.0 0.0
 0.0 0.0 0.0
 0.0 0.0 0.0
 0.0 0.0 0.0
 0.0 0.0 0.0
```
